# Adaptive Peer Tutoring and Insights From a Neurooncology Course

**DOI:** 10.2196/48765

**Published:** 2023-10-06

**Authors:** Burak Berksu Ozkara, Mert Karabacak, Zeynep Ozcan, Sotirios Bisdas

**Affiliations:** 1 Department of Neuroradiology MD Anderson Cancer Center Houston, TX United States; 2 Department of Neurosurgery Mount Sinai Health System New York, NY United States; 3 Cerrahpasa Faculty of Medicine Istanbul University-Cerrahpasa Istanbul Turkey; 4 Lysholm Department of Neuroradiology The National Hospital for Neurology and Neurosurgery University College London NHS Foundation Trust London United Kingdom

**Keywords:** COVID-19, distance learning, medical education, mentoring, peer teaching, web-based tutoring

## Abstract

Peer teaching in medicine is a valuable educational approach that benefits students and tutors alike. The COVID-19 pandemic has significantly impacted the advancement of remote education in the medical field. In response, the Cerrahpasa Neuroscience Society organized a web-based, volunteer-based peer tutoring program to introduce students to central nervous system tumors. This viewpoint examines our peer mentoring experience in medical education. We discussed how we shaped the course, its positive effects, and the flexible nature of the course, which brought medical students from different regions together. In addition to evaluating academic results, we examined the social relations made possible by this unique teaching method by analyzing student feedback and test scores. Finally, we discussed the promise of global web-based mentoring, highlighting its significance in the dynamic and global context of medicine.

## Background

Peer teaching in medicine is an increasingly recognized and valuable educational approach that benefits both students and tutors [1]. Using understandable language and relevant examples, this teaching strategy creates a stimulating and productive learning environment for medical students by leveraging the power of shared cognitive abilities and similar knowledge levels highlighted in the cognitive congruence hypothesis [2]. Students can gain insights into potential pitfalls and challenges by observing and discussing their peers’ mistakes and misconceptions, allowing them to fine-tune their own learning strategies. Moreover, students develop essential communication and teamwork skills through collaboration, which are critical for success in the health care field [3]. Furthermore, positive learning environments enhanced by peer interactions benefit medical education, as social congruence theory suggests that peers and near-peers are less threatening and better understand the stresses of the curriculum [1]. Another advantage of medical student peer education is that it provides relatable role models for professional development [1]. Tutors benefit significantly from peer education in medicine as well. Teaching others has been shown in studies to improve retention of material because it requires active engagement and a deeper understanding of the subject matter [4]. Tutors are likely to cultivate leadership abilities, which might be further enriched by their involvement in the course’s administrative facets.

The COVID-19 pandemic has significantly impacted the advancement of remote education in the medical field. As social distancing and lockdowns became prevalent worldwide, institutions quickly adapted to web-based learning platforms to ensure continuity in medical education [5]. This shift increased access to educational resources and sparked the development of novel teaching methods, such as simulations [6-8].

As members of the Cerrahpasa Neuroscience Society, we diligently contributed to the advancement of remote education during this challenging period. Throughout the pandemic, we organized fully remote webinars [9], student-led web-based journal clubs [10], and, finally, a web-based peer tutoring program. Instead of focusing on exam preparation or grade improvement, our first peer tutoring program aimed to introduce students to nervous system tumors. As a result, the course was entirely volunteer based, with tutors who were genuinely interested in and knowledgeable about the subject offering fresh insights into understanding the topic more thoroughly. To maintain a dynamic and responsive learning environment, tutors prioritized incorporating feedback into their planning processes, which was then used to refine the structure of subsequent sessions. In this viewpoint, we tackle an exploratory journey through the application of peer tutoring in medical education. We share the developmental process, positive impact, and practical flexibility of a peer-tutored course that connected medical students across Turkey, based on our experiences as tutors and organizers. We intend to assess not only the possible academic benefits but also the social advantages realized through this unique educational model through a detailed examination of tutee feedback, quiz results, and the personalized approaches adopted by the tutors. As we delve into the facets of this program, we highlight the broader potential for remote international mentorship, emphasizing its resonance with today’s ever-changing and interconnected medical landscape.

## Course Design, Implementation, and Assessment

The Cerrahpasa Neuroscience Society is a student-led organization that was founded in 2018 at the Cerrahpasa Faculty of Medicine, Istanbul University-Cerrahpasa. The Cerrahpasa Faculty of Medicine is an Istanbul-based public medical school that is one of Turkey’s oldest and most prestigious medical schools. The first 3 years of medical school at the Cerrahpasa Faculty of Medicine are preclinical, with clinical clerkships taking place in the 4th and 5th years. The sixth and final year is a pregraduate internship year.

A comprehensive neuroscience course, “Pathology and Radiology of Nervous System Tumors,” was scheduled to run from March 16 to May 11, 2021. Participants had to meet 2 requirements for earning a completion certificate: attend at least 80% of the classes and score a minimum of 80% on the final exam. The course was led by 2 experienced fifth-year medical students (BBO and MK), who served as the president and vice president of the Cerrahpasa Neuroscience Society. The course was divided into 5 lectures, with MK teaching the pathology topics and BBO covering the radiology aspects. We divided the lectures into five titles: (1) gliomas, (2) meningiomas and peripheral nervous system tumors, (3) central nervous system metastasis and primary central nervous system lymphoma, (4) childhood brain tumors, and (5) other nervous system tumors.

The program was open to applications from medical students of any grade and school due to the web-based format of events, which allowed a wide range of attendees. Attendance at the lessons, completion of quizzes, and acquisition of the certificate were provided at no cost. The course was promoted through the Cerrahpasa Neuroscience Society’s newsletter subscription, as well as the society’s website and social media platforms.

The 5 lectures were given over 9 weeks, with 2 weeks between the lectures. All lectures were held on the same weekday, starting at 5 PM on the Google Meet (Google LLC) platform. Lectures lasted approximately 2 hours, including a quiz and feedback. A few days before each session, participants would receive an email with resources to study beforehand to become familiar with the content of the week’s lecture. These emails would include both the English and Turkish versions of the resources, as well as the option to view the material in a simplified or detailed format. Given that not all tutees were at the same level of medical school and that many had not yet studied nervous system tumors, this was critical in preparing them for the lesson.

The first 4 sessions shared a consistent format: the material, which focused on a specific tumor subgroup, began with an explanation of the pathology from macro- to micropathology. To solidify understanding, emphasis was placed on visual materials and numerous examples. Next, the second tutor delved into the same topic through the lens of radiology, outlining how radiologic images should be interpreted. The second part of each session followed a similar lecture style. Notably, the first lecture centered on defining terminology and techniques in radiology, establishing a foundation for subsequent sessions. After the lectures, tutees tackled related medical cases under the tutors’ guidance. Students solving cases either volunteered or were selected by the tutors in the absence of volunteers.

Each session concluded with a 10-question multiple-choice quiz on the lecture content. The quiz encompassed both visual and verbal case questions, drawing from the provided resources and lecture information. Following the quiz, participants were presented with a feedback form to evaluate the tutors. This form incorporated a 5-point Likert scale to assess specific criteria, as well as open-ended questions designed to gather insights on strengths and weaknesses in order to improve future sessions (Table 1). Attendance was tracked based on the quiz and feedback form completion.

**Table 1:** Feedback form distributed to students after every lesson.

Question number	Question	Question type
1	“Preparation for the lesson”	5-point Likert scale
2	“Specifying the goals of the lesson”	5-point Likert scale
3	“The organization of the content”	5-point Likert scale
4	“Command of the subject matter”	5-point Likert scale
5	“Maintaining interactivity in the lesson”	5-point Likert scale
6	“Feedback given to the interacting students”	5-point Likert scale
7	“Maintaining the interest in the topic throughout the lesson”	5-point Likert scale
8	“Specifying the takeaway points”	5-point Likert scale
9	“What do you think was well done regarding the lesson?”	Open ended
10	“What would you like to see done in the upcoming lessons?”	Open ended
11	“If you have any additional comments or questions, please write them.”	Open ended

Although the fifth lecture was initially planned to follow the same structure, the tutors opted for a fully interactive session, with each participant solving at least one medical case. This revision-style approach, prompted by numerous suggestions, served as a comprehensive course review. Consequently, the final tumor subgroup lecture, which was not group-specific, was replaced with a recap of previous sessions through medical case resolution.

A total of 2 weeks after the lectures concluded, tutees with over 80% attendance received an exam question sheet through email and were asked to submit their answers within an hour. On evaluating the responses, tutees who scored above 80% were awarded course certificates.

This study was conducted in line with the principles of the Declaration of Helsinki. All participants provided informed consent after being fully informed about the lectures’ and surveys’ purpose and benefits. Ethical approval was deemed unnecessary by the institutional board because the survey responses were anonymous and stemmed from the Cerrahpasa Neuroscience Society’s peer-tutored courses, which were conducted remotely and independently of the university.

A total of 65 students from various medical schools throughout Turkey enrolled in the course. The Cerrahpasa Faculty of Medicine was the most represented institution, accounting for half of the participants.

The number of attendees gradually decreased over time, with 35 participants in the first lecture and only 12 in the final session. SPSS Statistics (version 26; IBM Corp) was used for descriptive statistical analysis. No bivariate analysis was conducted. The average quiz score improved throughout the course, with the exception of the final quiz, which had the lowest average grade (61.9 out of 100).

**Figure 1:**
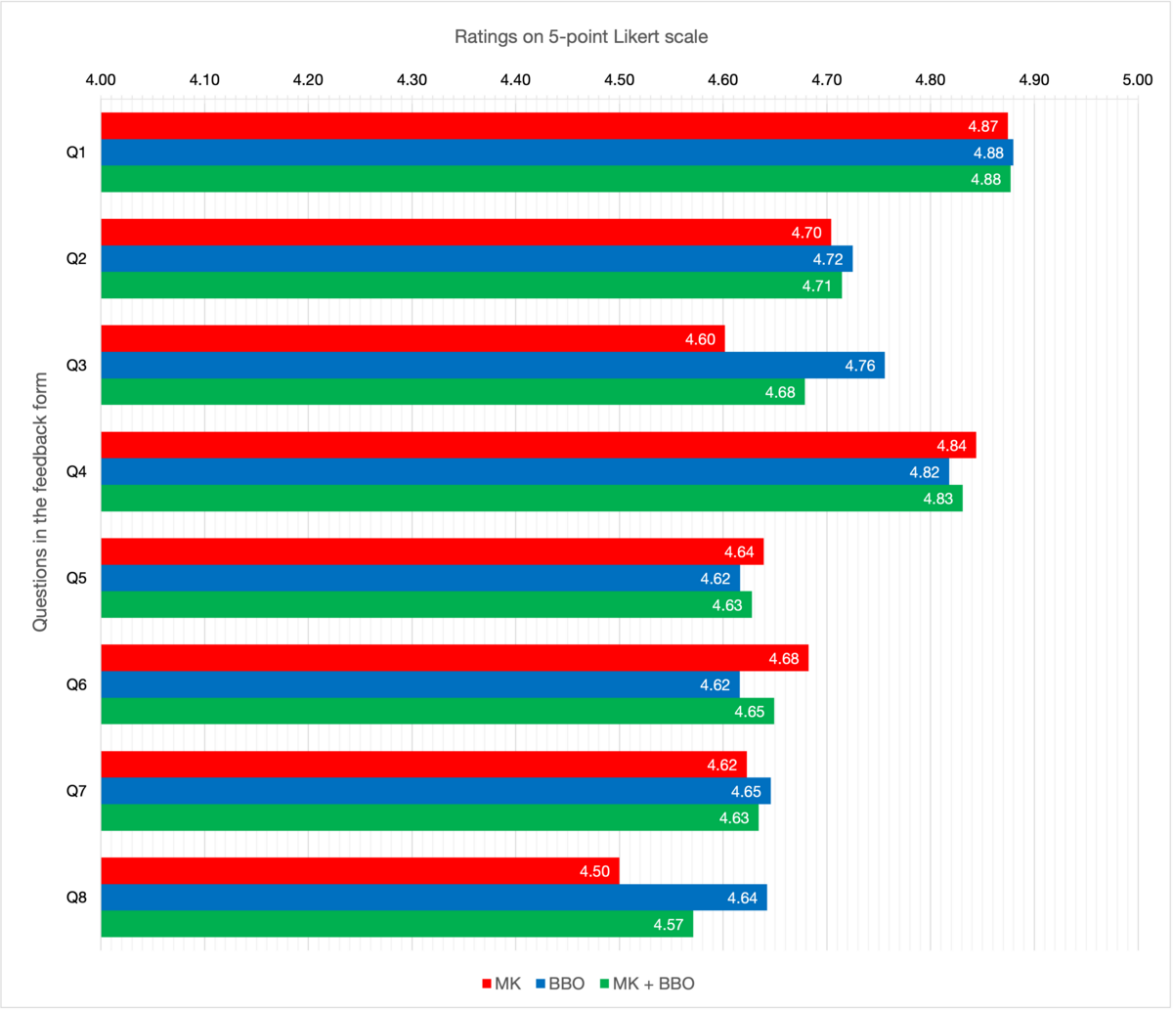
Mean tutor evaluation scores for each question, with ratings using a 5-point Likert scale (1=very poor and 5=excellent). The corresponding questions for the question numbers are given in Table 1. BBO: Burak Berksu Ozkara; MK: Mert Karabacak; Q: question.

**Figure 2:**
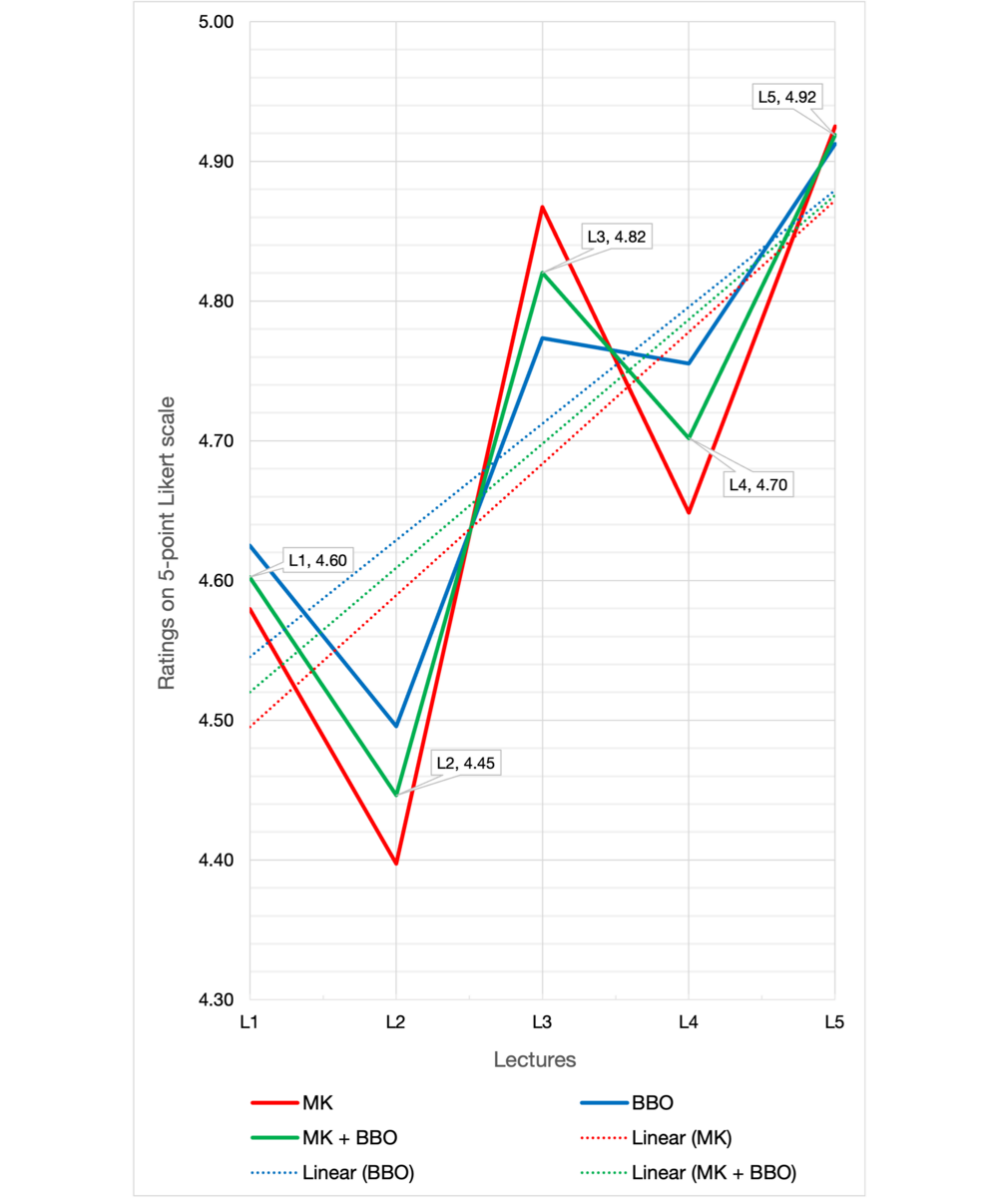
Mean tutor assessment scores for each session, listed in sequential order. Ratings are derived from a 5-point Likert scale (1=very poor and 5=excellent). BBO: Burak Berksu Ozkara; L: lecture; MK: Mert Karabacak.

Figure 1 displays the average scores of the tutors based on Likert-type questions after all lectures. Meanwhile, Figure 2 illustrates the average scores of the tutors for individual lectures, also based on Likert-type questions. Over the course, the highest average tutor rating was 4.88, which corresponded to “preparation for the lesson.” In contrast, the lowest average rating was 4.57, associated with “specifying the takeaway points.” The average of the 8 ratings for each tutor after each lecture fluctuated and did not display a consistent pattern.

Open-ended questions revealed that tutees primarily requested the inclusion of medical cases in the lectures. This suggestion was made by various tutees across multiple sessions, with the frequency decreasing as tutors incorporated more cases into their lectures. In earlier sessions, tutees requested more text-heavy slide organization and a slower pace. Once these needs were addressed, such feedback was no longer received. Over time, the number of suggestion-based comments decreased while expressions of appreciation for the tutors increased.

Out of the 7 tutees who submitted answers to the final exam, all surpassed the 80% threshold and qualified for a completion certificate. Comparing the quiz averages of these 7 tutees with the 28 who did not complete the course, the former group consistently achieved higher average scores in each quiz.

## Bridging Gaps in Pandemic Times

The COVID-19 pandemic has significantly impacted education, leading to obstacles in delivering quality instruction for medical students and exacerbating feelings of social isolation due to physical distancing. We introduce a peer-tutored course designed to not only enhance learning but also promote social interaction among participants. The responses from the participants suggested that the tutors may have fulfilled their roles satisfactorily, potentially creating a course that could have bridged the geographical and social gaps between a diverse group of Turkish medical students. During a period when quarantine may have contributed to feelings of isolation or had a negative impact on the psychological health of medical students, this initiative may have served as more than an educational tool, possibly fostering a sense of community and connection [11]. In doing so, it provides a valuable platform for these students, who are among the groups most affected by self-quarantine measures [12]. A student said,

As someone who felt quite alone during the pandemic, this course was a relief.

## Tailoring Lessons Through Feedback

Throughout the course, tutors maintained a dynamic lecturing style that readily adapted to students’ needs. A chronological analysis of the weekly feedback reveals that tutors diligently reviewed the scores and comments using them to tailor subsequent sessions. For example, after the first week where tutees reported insufficient interaction, tutors enhanced interactivity, leading to open-ended feedback that praised the increased engagement and usefulness of the case-solving sessions. In response to the subsequent week’s criticism of lesson organization, tutors revamped the third session’s slideshow by incorporating text alongside images, as suggested by the tutees. A tutee said,

As we previously desired, examining a large number of radiology images has been very positive for us, especially in terms of ingraining the pathologies we have reviewed into our minds. Discussing the finer points of radiological examinations has also been very beneficial for us. Thank you for taking our feedback into consideration.

This strategy and the flexibility of the lesson plans demonstrated how minor adjustments made in class can potentially boost student satisfaction. Having peer students as tutors and designing the course to be entirely volunteer based, naturally, may have allowed this flexibility to be practiced more smoothly. Nonetheless, there could be a lesson to take away from this demonstration, considering the integration of student feedback and requests within the application of the curriculum in medical school.

## Grades, Engagement, and Feedback

On examining the quiz averages, a general increase in topic comprehension may not be a coincidence. From our perspective, this improvement is likely attributable to the students who completed the program, likely due to their program satisfaction and success. The gradual grade progression is not observed on the final exam, which may be attributed to the detailed qualities of the questions covering the entire course. Nonetheless, this overall improvement should not be overlooked, as it may suggest that students are benefiting from a supportive environment. According to the test results, consistent participation may correlate with higher grades. The contrast between the 7 mentees who completed the course and the 28 who did not reveals the potential benefits of constant engagement and feedback. While we recognize that these results may be influenced by the individual characteristics of the students who chose to complete the course, our observations indicate a possible relationship between student engagement, feedback, and learning outcomes. While the limited number of students constitutes the primary limitation of our observations, it should not diminish the potential relevance of these insights. Additional research could strengthen these insights, revealing more about the interplay between these educational components.

## Peer Tutoring: Enhancing Education and Professional Growth

Despite the absence of empirical data, we, as tutors and authors of this paper, would like to emphasize the substantial benefits we have derived from the tutoring approach delineated by Ten Cate and Durning [1]. Considering that physicians must assume the role of educators, our tutoring experiences have significantly enhanced our preparedness and enthusiasm for this critical aspect of our profession. In addition to tutoring, our comprehensive involvement in organizing and administering the course from inception to completion has cultivated our leadership abilities and bolstered our confidence. Furthermore, this experience has solidified our commitment to prioritizing education as a fundamental component of health care. Currently, we continue to mentor several students from the course, an ongoing relationship that has allowed us to refine and augment our supervisory skills. Additionally, we believe that this experience enhanced our understanding of the topics we covered. Beyond the personal and professional growth, we have experienced, we believe that this peer-teaching model has positive implications for the education process itself. Peer teaching offers students a unique perspective on subject material as compared with a traditional curriculum. Tutors frequently draw on their own learning experiences, making it simpler for tutees to grasp the subject matter [13-15]. Having already mastered the core concepts, tutors can effectively guide their peers in focusing on what truly matters. We believe that these insights and experiences are not just theoretical concepts but tangible benefits that we have personally witnessed and experienced through our involvement in peer tutoring.

## Global Mentoring

After the COVID-19 pandemic, the normalization of remote communication paved the way for a more interconnected world, possibly allowing international remote mentors to play an increasingly important role in student education. These global mentors, who come from various cultural and professional backgrounds, can offer students invaluable insights and guidance that cross geographical boundaries, fostering a deeper understanding. Students can gain a fresh perspective on their academic pursuits while also developing the skills needed to navigate the interconnected and rapidly evolving world they will eventually enter as professionals by tapping into the wealth of knowledge that remote mentors bring to the table. As members of the Cerrahpasa Neuroscience Society, we are grateful to be receiving support from author SB, whose inspiration led us to establish this peer tutoring course.

## Importance of the Course

In the ever-changing landscape of web-based medical education, it is essential to emphasize what makes our peer-taught course distinctive. To the best of our knowledge, web-based radiology-pathology courses tailored to the Turkish context are scarce. Our course is the first ever free radiology-pathology correlative course taught by students in Turkey. This distinction is significant, particularly in light of the limited context-appropriate web-based resources available to Turkish medical students, especially while many Turkish medical students perceived themselves as having inadequate radiology skills [16]. Moreover, the fully peer-taught model of our course is a novel approach in our region. This method gained even more importance during the challenging times of the COVID-19 pandemic. While some may believe that our course’s research foundation and methods are comparable to those of other global web-based teaching initiatives, it is essential to consider the context in which our course was conceived and implemented. This paper’s primary objective is not to delve into methodologies but to highlight the innovation in addressing a distinct gap for Turkish medical students during a global pandemic by using peer-led strategies.

## Future Directions and Limitations

Our experiences with peer tutoring, though enlightening and inspiring, are not without their limitations. This paper’s conclusions are primarily based on qualitative feedback and subjective observations, as opposed to a comprehensive empirical framework. This lack of extensive data limits our ability to make broad generalizations; therefore, this paper is classified as a viewpoint piece emphasizing personal insights and contextual interpretation. The decreasing number of students was another concern. This could be due to a variety of factors. Even if the web-based activities were helpful, the pandemic made many people feel exhausted [17]. Even though our course was organized to help students, it was challenging due to the difficult medical topics covered. As attendance was voluntary, some students may not have felt pushed to remain. Technical difficulties with web-based sessions may have discouraged some. Last, personal issues may have impacted their decision to continue during this difficult time. In addition, the program’s voluntary nature may have resulted in selection bias, as more motivated students were more likely to participate, potentially skewing the results. Future research could address these limitations by integrating better evaluation methods, involving various educational settings, and increasing the number of participants. Examining the long-term effects of such peer-tutoring initiatives on tutors and tutees and the integration of similar approaches into traditional curricula could provide a deeper understanding of the numerous advantages of these educational techniques.

## Data Availability

The data sets generated during and/or analyzed during this study are available from the corresponding author on reasonable request.
